# Locomotion Mode Transition Prediction Based on Gait-Event Identification Using Wearable Sensors and Multilayer Perceptrons

**DOI:** 10.3390/s21227473

**Published:** 2021-11-10

**Authors:** Binbin Su, Yi-Xing Liu, Elena M. Gutierrez-Farewik

**Affiliations:** 1 KTH MoveAbility Lab, Department of Engineering Mechanics, KTH Royal Institute of Technology, 10044 Stockholm, Sweden; binbins@kth.se (B.S.); lyixing@kth.se (Y.-X.L.); 2 Department of Women’s and Children’s Health, Karolinska Institute, 17177 Stockholm, Sweden

**Keywords:** critical gait events, locomotion mode, exoskeleton control

## Abstract

People walk on different types of terrain daily; for instance, level-ground walking, ramp and stair ascent and descent, and stepping over obstacles are common activities in daily life. Movement patterns change as people move from one terrain to another. The prediction of transitions between locomotion modes is important for developing assistive devices, such as exoskeletons, as the optimal assistive strategies may differ for different locomotion modes. The prediction of locomotion mode transitions is often accompanied by gait-event detection that provides important information during locomotion about critical events, such as foot contact (FC) and toe off (TO). In this study, we introduce a method to integrate locomotion mode prediction and gait-event identification into one machine learning framework, comprised of two multilayer perceptrons (MLP). Input features to the framework were from fused data from wearable sensors—specifically, electromyography sensors and inertial measurement units. The first MLP successfully identified FC and TO, FC events were identified accurately, and a small number of misclassifications only occurred near TO events. A small time difference (2.5 ms and −5.3 ms for FC and TO, respectively) was found between predicted and true gait events. The second MLP correctly identified walking, ramp ascent, and ramp descent transitions with the best aggregate accuracy of 96.3%, 90.1%, and 90.6%, respectively, with sufficient prediction time prior to the critical events. The models in this study demonstrate high accuracy in predicting transitions between different locomotion modes in the same side’s mid- to late stance of the stride prior to the step into the new mode using data from EMG and IMU sensors. Our results may help assistive devices achieve smooth and seamless transitions in different locomotion modes for those with motor disorders.

## 1. Introduction

Powered rehabilitative devices have been designed to aid people with neurological disorders who present with symptoms, such as muscle weakness and poor coordination. Wearable assistive devices can apply torque at the ankle, knee, and hip joints during walking using advanced sensing and control strategies, for example, controlling plantar flexion torque [1,2,3], controlling knee torque [4,5,6], and setting knee equilibrium points [7]. Such powered devices are also used in various locomotion modes other than level-ground walking, such as ramp walking and stair ascent/descent [8,9]. As gait kinematics vary in different locomotion modes, the robotic devices must discriminate between these modes to ensure seamless and natural transitions.

As such, these devices normally include a high-level locomotion mode classifier and a mid-level state machine to assert appropriate torques for each mode [10]. Locomotion mode classifiers are commonly built with machine learning (ML) algorithms, such as support vector machine (SVM), linear discriminant analysis, and artificial neural networks (ANN), using as input data from a set of sensors, such as electromyography (EMG) sensors and inertial measurement units (IMUs) [11,12,13,14].

These wearable sensors are relatively inexpensive and can be donned outside of a lab environment. They can also feasibly be incorporated into exoskeleton hardware. ML is an effective tool to extract prominent features and make statistical inferences from these data. The integration of IMUs, EMG sensors, and ML has become an increasingly common approach to achieve high classification performance in identifying locomotion modes and transitions [15,16,17,18,19].

Mahoney et al. [20] used ANN models to classify several locomotion modes, such as walking, jogging, and running. They trained the model with accelerometer data, gyroscope data, and a combination of both. A common practice to improve classification accuracy has been to apply majority voting that adds time-history information [21]. Huang et al. [21] used SVM to identify locomotion modes in subjects with a knee prosthesis with inputs from EMG signals and mechanical sensors (six-DOF load cells). Each locomotion mode was identified by a majority voting of multiple binary classifiers. Liu et al. [22] enhanced a muscle synergy-inspired method of detecting movement intentions by using fused wearable sensor data and majority voting.

Similarly, these locomotion mode classifiers are often paired with gait phase or gait-event detection, thus, providing important information during locomotion about critical events, such as foot contact (FC) and toe off (TO). A powered prosthesis or exoskeleton must not only identify the current locomotion modes but must also correctly predict upcoming ones before these critical events, i.e., the locomotion mode transition must be predicted prior to the critical event that marks the onset of the next locomotion mode, so that the powered device generates the appropriate gait pattern.

Locomotion modes are typically labeled between the FC and TO, which, in turn, are often identified through motion capture systems [23], load cells [24] and IMUs [25,26]. However, motion capture systems generally require a specialized lab, which is not always feasible. Force sensors, such as foot switches and foot pressure insoles, have limited life spans, are sensitive to the insole interface, and may not always be suitable for daily life applications [27,28,29].

IMUs have been widely used to predict locomotion modes due to their portability, durability, reliability, and low energy consumption [30,31,32]. However, IMU application to detect gait phases and events has often been based on threshold-based methods. For example, FC and TO has been extracted from shank angular velocity in the sagittal plane as follows: FC has been defined as minimum velocity following peak velocity in swing, and TO has been defined as the halfway point between zero-crossings that indicates a change in the shank direction [25]. However, these threshold-based definitions involve manually setting a set of rules based on certain characteristics of gait phases and gait events.

Machine learning approaches are popular for predicting gait phases and events due to their ability to learn and recognize hidden patterns from data without being explicitly programmed. Attal et al. proposed a hidden Markov model to segment the gait cycle into different gait phases without a manual labeling process [33]. Vu et al. [34] proposed an exponentially delayed fully connected neural network to segment a full gait cycle discretized into 1% intervals.

Their method provided prosthetic devices with better control by, for example, signaling to the spring to store energy during mid-stance and the motor to release positive energy during pre-swing. Ledoux used 3 machine learning algorithms to classify sensor signals into stance and swing phases, detecting 100% of the gait events within an average of 2% stride time [35]. In a previous study, we distinguished five gait phases using a neural network based on the acceleration, angular velocity, and magnetic field intensity from IMUs attached on the thighs, shanks, and the feet, achieving approximately 97% accuracy during an offline evaluation of gait phase recognition [36].

Locomotion mode and gait-event prediction have been individually explored with machine learning methods. It can be beneficial and convenient to integrate locomotion mode prediction and gait-event identification into one machine learning framework, as gait-event detection is often a vital part of locomotion mode prediction. In this study, two multilayer perceptrons (MLPs) were proposed to predict locomotion mode transitions using wearable sensors, namely EMG sensors and IMUs. In the first MLP, critical gait events, i.e., FC and TO, were identified with IMU signals. In the second MLP, the locomotion mode transitions were predicted using EMG and IMU signals based on the FC and TO events extracted from the first MLP.

## 2. Methods

### 2.1. Participants

Eight subjects without any musculoskeletal disorders or recent lower-extremity injuries (four men and four women, mean ± standard deviation age: 28.3 ± 1.4 years old, height: 1.71 ± 0.09 m, and body mass: 62.3 ± 12.7 kg) participated. The experiments were approved by the Swedish Ethical Review Authority (Dnr. 2020-02311). All subjects provided written consent.

### 2.2. Experimental Protocol and Data Collection

Surface EMG sensors were placed according to SENIAM recommendations [37] on eleven muscles on the right limb: the gluteus maximus, gluteus medius, rectus femoris, vastus medialis, vastus lateralis, semitendinosus, tibialis anterior, gastocnemius medialis and lateralis, soleus, and peroneus longus; and nine muscles on the left limb: the gluteus medius, rectus femoris, vastus medialis, vastus lateralis, semitendinosus, tibialis anterior, gastrocnemius medialis, soleus, and peroneus longus. IMU sensors were placed on the left and right thighs, shanks, and feet, and one on the pelvis.

**Figure 1:**
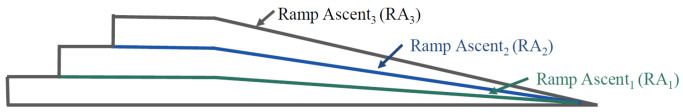
The ramp-stairs module with three adjustable inclination angles.

Foot switches, placed under the toe and heel of each foot, were used to detect FC and TO events for the training samples. EMG and IMU signals were sampled at 2000 and 286 Hz, respectively. IMU signals were then interpolated to have the same frequency with EMG signals. A tailored ramp-stairs module (Figure 1) was specifically crafted for the experiment. This module had three adjustable inclination angles (4${}^{\circ }$(A1), 8${}^{\circ }$(A2), 12${}^{\circ }$(A3)) and stairs with an 18 cm rise and a 28 cm run.

For the stepping-over-an-obstacle task, subjects were instructed to walk on level ground and then step over a box (15 cm high and 30 cm wide) with their right leg and then proceed walking. For other locomotion modes, subjects started with level-ground walking, walked towards the ramp-stairs module, transitioned to stair and/or ramp ascent, continued walking on the middle platform, performed ramp and/or stair descent (always the opposite as the approach), proceeded with level-ground walking, and finally stopped to a standing position. For the sake of minimizing terminology, even though consecutive steps in the same locomotion mode, for instance, consecutive steps of stair ascent, are not actually transitions, they are referred to as such in this study.

Thus, in the experiment, there were seven transitions from walking, six transitions from ramp ascent, and six transitions from ramp descent. The transitions from level-ground walking (W) are: level-ground walking (W→W), stair ascent (W→SA), ramp ascent (W→RA), stair descent (W→SD), ramp descent (W→RD), stepping over an obstacle (W→O), and stopping (W→ST). The ramp ascent transitions are further discretized according to the three inclination angles A1–A3, i.e., from ramp ascent to: ramp ascent at the same angle (RA1→RA1, RA2→RA2, and RA3→RA3) and to level ground walking (RA1→W, RA2→W, and RA3→W). Similarly, ramp descent transitions are from ramp descent to: ramp descent at the same angle (RD1→RD1, RD2→RD2, and RD3→RD3) and to level ground walking (RD1→W, RD2→W and RD3→W). Each task was repeated at least 10 times. During data collection, all subjects were encouraged to walk at a self-selected, comfortable speed and to try to maintain this speed as closely as possible.

### 2.3. Data Processing

EMG signals were first rectified to obtain only positive values. The rectified signals were passed through a fourth-order Butterworth high pass filter with a cutoff frequency of 20 Hz to remove motion artifacts [38] and then through a fourth-order Butterworth low pass filter with a cutoff frequency of 200 Hz to eliminate high-frequency noises [39]. Linear acceleration and angular velocity in three directions from the IMUs were low-pass filtered at 20 Hz with a Butterworth filter [40]. The Butterworth filters have zero-phase delay properties such that the filtering does not result in a temporal shift. The IMU data were normalized by the mean and standard deviation (Z score normalization, i.e., each value minus the mean, and then divided by the standard deviation) of each degree of freedom.

Since there is no exact instance for discriminating the transition between two locomotion modes, we identified TO as the critical gait event. For all transitions, the ’from’ transition was the one in the stance phase prior to the transition, and the critical event to detect was TO just before the same leg transitioned to (took a step into) a new mode.

For the first-layer classifier, the input was a 42-element vector of concatenate features of linear acceleration and angular velocity in three directions, and the output was the gait event, i.e., FC, TO, or neither/others. FC and TO were identified by the foot switches at the instant they registered contact (FC) or lack thereof (TO). Others were labeled when the instances were not FC or TO. After training, the foot switches were removed, and the output of the first classifier was used for labeling the locomotion mode transitions in the second-layer classifier.

For the second-layer classifier, the input was the EMG and selected IMU signals from the first classifier. Sliding analysis windows were used to segment the input for continuous classification decision making. The length of the sliding windows was 100 ms, and the window increment was 12 ms. The inputs were concatenated into a 62-element feature vector for each analysis widow. The output is one of the locomotion mode transitions, e.g., W→RA.

### 2.4. Multilayer Perceptron (MLP)

Two MLPs were created—the first to detect FC and TO and the second to predict locomotion mode transitions based on the detected critical events from the first MLP. An MLP is a universal approximator that can ideally match the input features to a label, given enough hidden units. The benefit of using two MLPs is the integration of a single learning mechanism that performs two different tasks. Each MLP is comprised of three layers. The first layer is the input layer, in which the features are used as the initial input. The middle layers are called hidden layers, each of which contains several hidden units. The third layer, the output layer, produces the desired output. Normally, each layer except the input layer performs a nonlinear activation function to capture the nonlinear data relationship.

The activation functions of the hidden and output layers are the Relu function and the softmax function, respectively. The network weights and biases were updated using batch gradient descent with adaptive moment estimation (Adam) optimization algorithm and categorical cross-entropy loss. The model’s hyperparameters were determined with the grid search approach, including the number of epochs, batch size, layers, and cells. The optimum model was then trained for 100 epochs with an early stop if the model performance did not increase in the consecutive 10 epochs. The best model for the first MLP has one hidden layer with 64 units using a batch size of 16.

The best model for the second MLP has one hidden layer with 128 units using a batch size of 64. The first MLP outputs the corresponding gait events, i.e., FC, TO, and Others directly while the second MLP used a voting strategy to predict the locomotion mode transition. The voting strategy means that the transition was determined with the most votes out of all the analysis windows within the predicted FC and TO in a gait cycle. The performance of the various classifiers was evaluated in intra-subject analyses via leave-one-out cross-validation (LOOCV) with 10 trials collected from each subject, i.e., nine trials were used to train the classifier, the remaining trial was used to test/detect, and then the process was iterated through all trials.

### 2.5. Performance Evaluation

The first MLP classifier detected FC, TO, and Others in all walking transitions, ramp ascent transitions, and ramp descent transitions. The second MLP classifier predicted the locomotion mode transitions within the interval of detected FC and TO in a gait cycle. Accuracy was computed as the proportion of correctly classified data in the total amount of data. In addition, the confusion matrix was computed to demonstrate the relationship between the actual classes and the predicted classes. Each row of the matrix represents the instances in an actual class, while each column represents the instances in a predicted class.

Accuracy can be a misleading metric for imbalanced data sets. Finally, the recall, precision, and F1 score were computed from the confusion matrix to determine how accurate the classifiers were out of the predicted classes and true classes. Recall is defined as the number of the correctly predicted class divided by the number of the true class. Precision is defined as the number of the correctly predicted class divided by the number of the predicted class. The F1 score is the harmonic mean of the recall and precision.

## 3. Results

The accuracy, recall, precision, and F1 score for each locomotion mode transition were averaged for the eight subjects. The accuracy always refers to the diagonal axis of the confusion matrix. The diagonal axis of the confusion matrix is exactly the same as the recall.

### 3.1. Performance in FC and TO Identification

**Figure 2:**
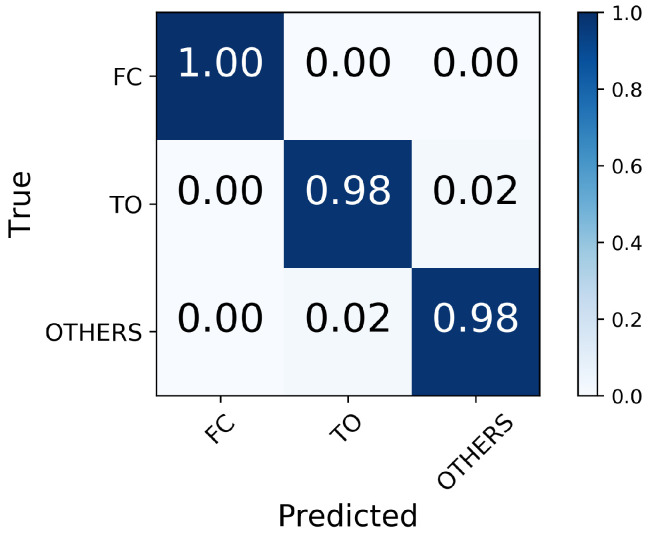
Confusion matrix for FC and TO identification.

**Figure 3:**
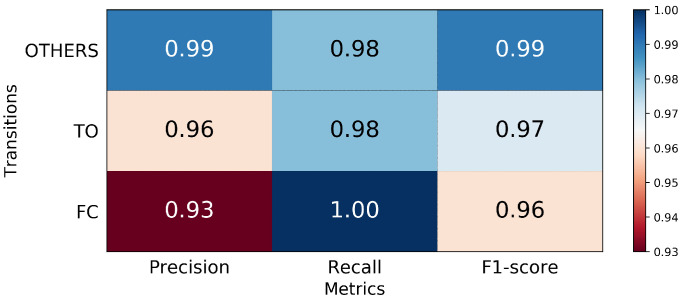
The precision, recall, and F1-score for FC and TO identification.

In the first classifier, the MLP accurately predicted 100% FC and 98% TO events. Misclassifications occurred only between the TO and Other events (Figure 2). While high recall scores can refer to the diagonal elements of the confusion matrix, the precision was also high in FC (93%) and TO (96%); this result indicates that the number of false positive predictions was small (Figure 3). The prediction time of FC and TO events is demonstrated in Table 1. The results were averaged across subjects and trials; the mean prediction time was 2.5 ms before FC and 5.3 ms after TO. Missed transitions were excluded from the calculation.

### 3.2. Performance in Predicting Transitions from Walking

**Figure 4:**
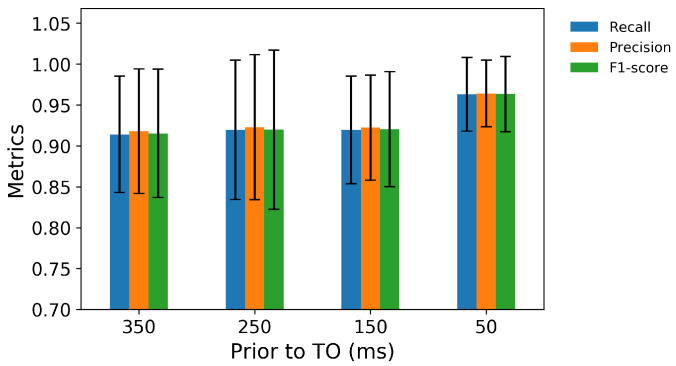
The precision, recall, and F1-score for predictions of transitions from level-ground walking 350, 250, 150, and 50 ms prior to TO.

**Figure 5:**
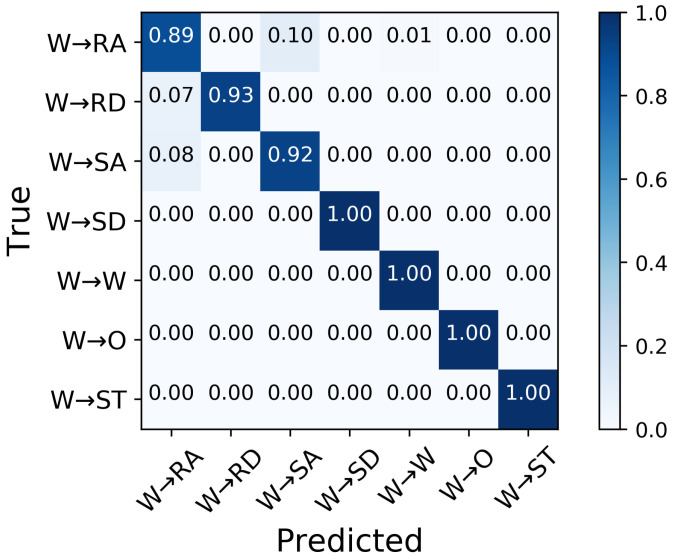
Confusion matrix of the average classification accuracy of predictions of transitions from level-ground walking 50 ms prior to TO.

In the second classifier for locomotion mode transition recognition, the MLP can predict the transitions from level-ground walking up to 350 ms prior to TO with over 90% aggregate accuracy (Figure 4). For predictions 50 ms prior to TO, the classifier achieved an aggregate accuracy of 96.3% in the seven transitions from level-ground walking, with 100% accuracy in W→SD, W→W, W→O, and W→ST transition and the lowest accuracy in W→RA (Figure 5).

**Figure 6:**
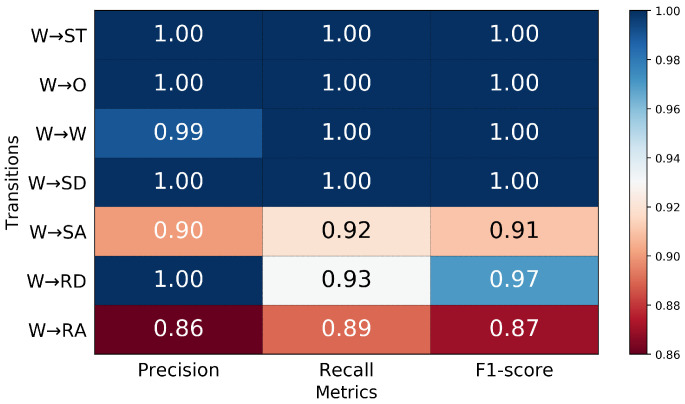
The precision, recall, and F1-score for predictions of transitions from level-ground walking 50 ms prior to TO.

The precision, recall, and F1-score of seven walking transitions are reported in Figure 6. The highest precision was achieved in W→ST, W→O, W→SD, and W→RD transitions; these transitions were correctly predicted with no false predictions. The remaining transitions from walking all achieved at least 86% precision, which indicated that the classifier was robust to type I errors. This robustness is the result of a small number of false positives that occurred when the classifier estimated a transition, but it did not actually occur.

### 3.3. Performance in Predicting Transitions from Ramp Ascent

**Figure 7:**
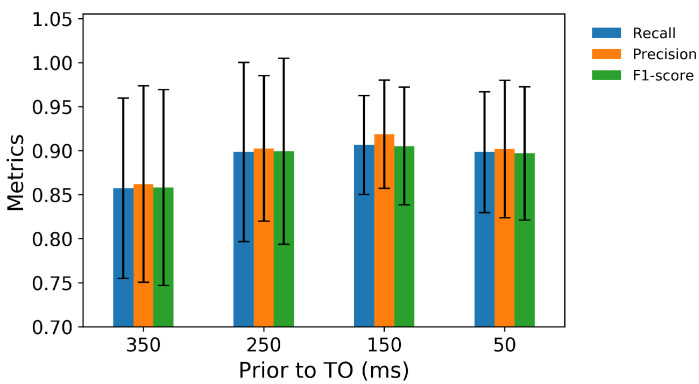
The precision, recall, and F1-score for predictions of transitions from ramp ascent 350, 250, 150, and 50 ms prior to TO.

**Figure 8:**
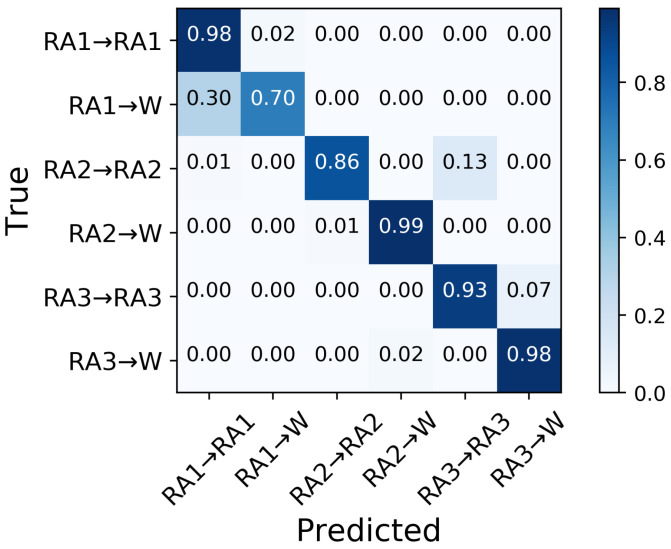
Confusion matrix of the average classification accuracy of the ramp ascent transitions 150 ms prior to TO.

**Figure 9:**
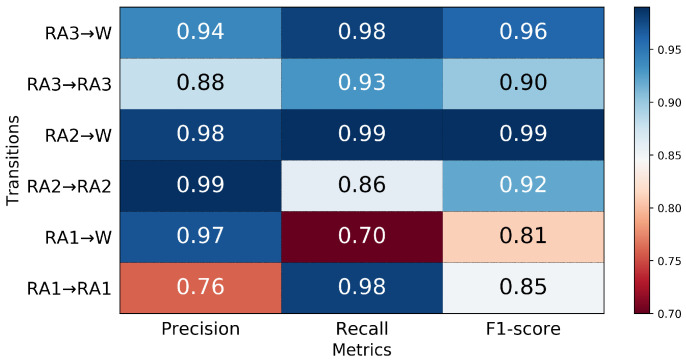
The precision, recall, and F1-score for transitions from ramp ascent 150 ms prior to TO.

In the ramp ascent mode, the classifier could overall identify RA prior to TO (Figure 7). Transitions from ramp ascent were predicted 150 ms prior to TO with an aggregate accuracy of 90.6% in the six transitions. The model correctly predicted RA2→W (99%), followed by RA1→RA1 and RA3→W (98% in both), RA3→RA3 (93%), RA2→RA2 (86%) and RA1→W (70%) (Figure 8). 30% of RA1→W transitions were misclassified, wherein all of them were predicted as RA1→RA1. These misclassifications are also reflected in Figure 9, indicating that the RA1→RA1 transition prediction had the lowest precision of 76%. The highest precision was achieved in the RA2→RA2 transition; 99% predicted RA2→RA2 transitions were true.

### 3.4. Performance in Predicting Transitions from Ramp Descent

**Figure 10:**
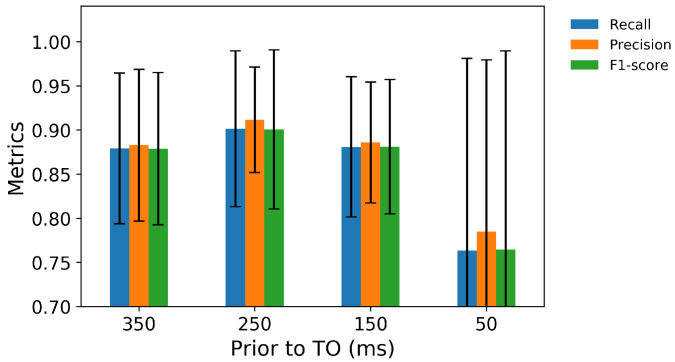
The precision, recall, and F1-score for predictions of transitions from ramp descent 350, 250, 150, and 50 ms prior to TO.

**Figure 11:**
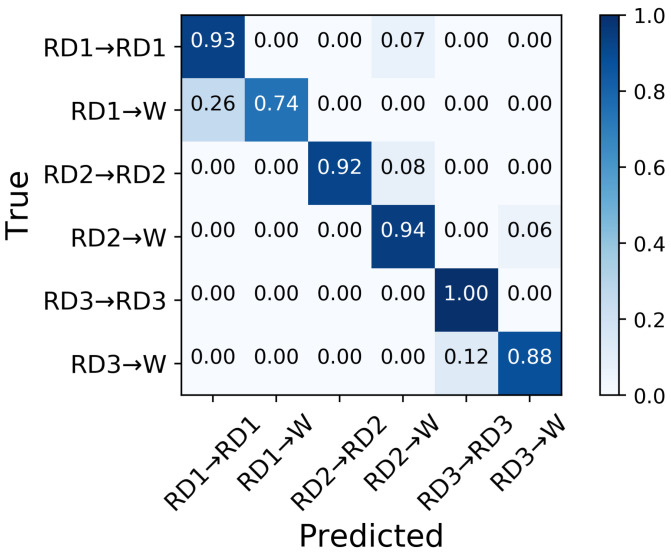
Confusion matrix of the average classification accuracy of predictions of transitions from ramp descent 250 ms prior to TO.

**Figure 12:**
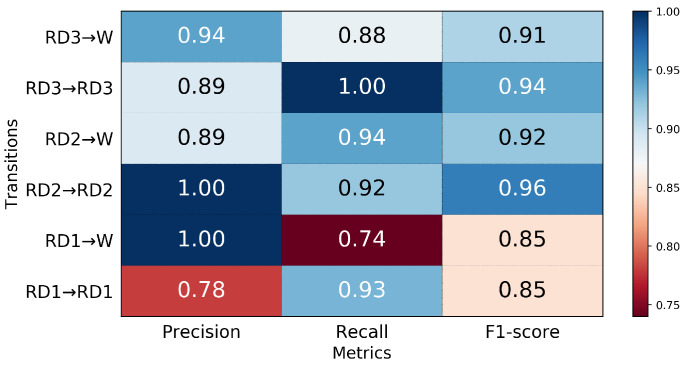
The precision, recall, and F1-score for transitions from ramp descent 250 ms prior to TO.

The six transitions from ramp descent were predicted with an aggregate accuracy of over 90% 250 ms prior to TO (Figure 10); RD3→RD3 was predicted with the highest classifier accuracy (100%) and RD1→W with lowest accuracy (74%). A total of 26% of RD1→W transitions were misclassified as RD1→RD1 (Figure 11), resulting in the lowest precision 78% in RD1→RD1 (Figure 12). The highest precision was achieved in RD1→W and RD2→RD2; all of these were predicted correctly.

## 4. Discussion

Movement patterns change as people transition from one terrain to another, and this can be challenging for people with motor disabilities. Predicting transitions between locomotion modes is important for developing new rehabilitation protocols and assistive devices (e.g., exoskeletons) tailored for each user. In this paper, we developed two MLPs that can continuously predict a variety of locomotion mode transitions based on critical gait events (FC and TO) detection.

The first layer MLP classifier trained with IMU data performed well in identifying critical gait events FC and TO; the FC was estimated correctly, and a small number of misclassifications only occurred between TO and other states. This finding is consistent with the observation by [41] who reported that TO occurs later in the gait cycle; hence, true TO is falsely classified as other phases. High precision was achieved in FC and TO, which means that only a fraction of predicted FC and TO are false. This agrees with the prediction time of predicted FC and TO before the true gait event, which reported a small time difference (2.5 ms and −5.3 ms for FC and TO, respectively) between the predicted and true events.

The mean time differences were very small compared to the time of a normal gait cycle [42]. Therefore, the predicted FC and TO can be reliably used to predict locomotion mode transitions in the second classifier. Note that the first MLP used sample-based data as inputs, and thus stride-to-stride frequency information was lost. Thus, the gait events were most likely detected based on the local maxima/minima and inflection points in the IMU data coinciding with these gait events. Huang et al. fused EMG and mechanical sensor signals and reported 99% accuracy in predicting the stance phase and 95% accuracy in predicting the swing phase.

They used ground reaction force and moments as inputs for the classifier and labeled gait phases based on the ground reaction force [14]. Our approach excludes the ground reaction force information in the input data. We attempted to use the MLP to extract the representation of the IMU data, which could be used to map the corresponding FC, TO, or neither of them. The high accuracy we found in detecting FC and TO (100% and 98%) indicates that the MLP was able to capture the mapping between the input features and the gait events.

Sabatini et al. proposed that gait events can be established from only the shank angular velocity in the sagittal plane. Their approach of detecting FC and TO required hand-tuned heuristics, and the performance was subject to degradation at high inclines. Our approach can possibly capture gait characteristics that are not specifically coded inside the MLP network; the MLP acted as a universal approximator that created a function to identify gait events from the acceleration and angular velocity of IMUs.

The second layer MLP classifier, trained with IMU and EMG data and segmented by FC and TO events detected in the first layer, showed high accuracy in predicting transitions from level-ground walking, ramp ascent, and ramp descent. It is worth highlighting that predicting transitions up to 350 ms before the TO means that, when a limb was in approximately mid-stance, the MLP could predict which mode that same leg would enter at its next step. For transitions from level-ground walking, the accuracy increased as the prediction time approached the critical TO event, i.e., during the late stance or pre-swing of the same side.

While the classifier predicted transitions from level-ground walking most accurately (96%) immediately (50 ms) prior to TO, the transitions were still predicted with over 90% accuracy less immediately (up to 350 ms) prior to TO, which, in many cases, will be between 0.73 and 0.77 seconds prior to the step into the new mode, given that the swing phase lasts, on average, 0.38 to 0.42 s [43]. These prediction times may be crucial for exoskeleton controllers to ensure smooth and safe changes into a different mode, as delays can be expected. An assistive device, thus, may have time to provide adequate foot clearance, increase the range of knee flexion, and prevent tripping over an obstacle, staircase, or incline.

In ramp ascent and ramp descent modes, the classifier could predict another step with the same inclination angles 250 ms prior to the TO with over 90% accuracy. In a recent study using a different classifier, the authors achieved an average of 95.1% accuracy in predicting transitions from level-ground walking [22]. The method in the current study was slightly more accurate (96.3%) with 100% accuracy in predicting W→SD, W→W, W→O, and W→ST transitions. Afzal et al. [44] reported 85% and 77.9% accuracy for predicting RA and RD, respectively, using a method that identifies muscle synergies in EMG data; 11.4% of RA and 17.9% of RD were incorrectly identified as level ground walking.

**Figure A1:**
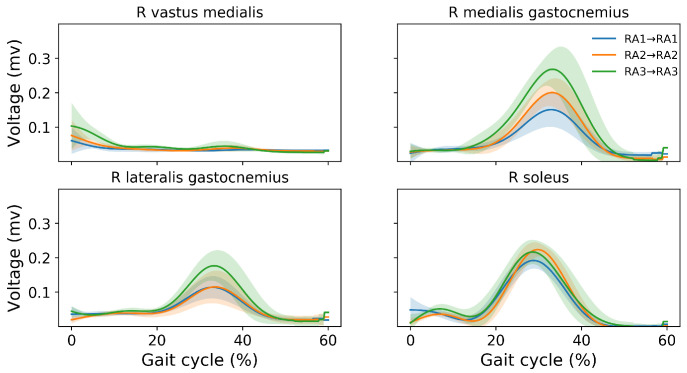
EMG recordings of the vastus medialis, medialis gastocnemius, lateralis gastocnemius, and soleus in the right limb under different ramp ascent transitions RA1→RA1, RA2→RA2, and RA3→RA3 of a representative subject.

The method in the current study displayed an aggregate accuracy of 92.3% and 95% for predicting RA→RA and RD→RD, respectively, at three inclination angles. The highest prediction accuracy was achieved for the steepest inclination angle A3. This can be attributed to the more unique gait features at greater angles than at less steep angles or level ground. For example, hip and knee flexion were usually greater at steeper ramp angles. Similarly, upon visual inspection, EMG signals tended to indicate higher muscle activation at higher inclination angles (Appendix A Figure A1): in particular, the vastus medialis, gastrocnemius, and soleus.

The overall successful prediction of transitions from one locomotion mode to another can be attributed to three factors. The first is the fusion of EMG and IMU sensor data that provides comprehensive information about segment movement and underlying muscle activation in anticipation of transitions to another locomotion mode. The second is the majority voting strategy that combines prediction from multiple analysis windows in one stance period. Since the EMG and IMU patterns are not entirely unique for different transitions, the disparity is only present within a specific interval between FC and TO; thus, the analysis windows can extract prominent differences between transitions and return the correct transition based on a majority voting. This strategy also offers lower variance in the predictions.

The third is the proper filtering that attenuates the low and high frequency noise components of EMG and IMU signals without introducing a temporal shift in the signals. Therefore, the early prediction before TO can be considered reliable and not subject to phase shift. It is worth mentioning that our method did not always predict transitions more accurately than the approaches proposed by Liu et al. [22] and Afzal et al. [44]; however, to the best of our knowledge, this is the first time that locomotion mode prediction and gait-event identification have been embedded into one framework. As the foot switches mainly worked as a tool for labeling data, they could be removed after the models had been trained. This process can solve potential problems with mechanical failures and false activation in foot switches in addition to eliminating the difficulty in putting foot switches on amputees or subjects with foot deformities.

Transitions to and from locomotion modes were predicted with high accuracy, particularly in W→SD, W→W, W→O, and W→ST, without relying on foot switches. Our findings indicate that force sensors can be absent in methods to predict locomotion mode, which led to high system robustness, as force sensors are sensitive to the skin and insole interface and are prone to mechanical failure.

There are some limitations in the study. The experiment involved only able-bodied subjects. Whether transitions can be predicted as accurately in persons with motor disorders, who are much more likely to be users of exoskeletons, remains to be studied. Predicting transitions between locomotion modes was based on the successful identification of critical gait events. Although our approach rarely missed a critical gait event, any missed gait event could undermine the performance in predicting a transition. In addition, this study adopted a data fusion strategy widely used in locomotion mode transition prediction.

We did not consider a feature selection procedure that could potentially lead to better performance. This study demonstrated that transitions between locomotion mode can be achieved using fused data from EMG and IMU signals; however, we did not aim to provide detailed explanations of why the performance varied among different transitions and what caused the difference. This remains a challenge for all machine-learning models.

The underlying factors that influence the performance of the classifier could be useful for developing automatic and seamless exoskeleton controllers. In addition, our analyses were performed offline with experimental data collected in the lab. The performance of transition prediction based on gait-event identification for subjects walking in ecological situations and in real-time warrants further study.

## 5. Conclusions

In this study, we integrated locomotion mode transition prediction and gait-event detection into one machine learning framework, consisting of two MLPs as well as fused EMG and IMU signals. The first MLP detected the critical FC and TO events with 100% and 98% accuracy, respectively. The timing of FC and TO events was within approximately 5 ms of the true events. This high accuracy in detecting FC and TO events contributed to the success of the second MLP in predicting transitions from one locomotion mode either to the same mode or to another using fused EMG and IMU signals as inputs.

The second MLP correctly identified transitions from level-ground walking, ramp ascent, and ramp descent with sufficient prediction time prior to the gait events, i.e., in midstance, late stance or pre-swing of the same side prior to the step into the new mode, with the accuracy increasing and approaching TO of the same side. Furthermore, our method was able to accurately predict consecutive steps in ramp ascent and descent at different inclination angles. The achieved results may aid in the design of assistive devices tailored for those with motor disorders affecting gait.

## Figures and Tables

**Table 1:** The prediction time (ms) of FC and TO before the true gait events. A positive value represents the predicted event prior to the true event, while a negative value indicates that the predicted event occurs after the true event.

Gait Event	Mean	Std	Min	25%	50%	75%	Max
FC (ms)	2.5	24.2	−40.0	−10.0	0.0	2.5	100.0
TO (ms)	−5.3	19.1	−70.0	−10.0	0.0	0.0	30.0

## Data Availability

Data available on request due to privacy and ethical reasons. The data presented in this study are available on request from the corresponding author.

## Abbreviations

The following abbreviations are used in this manuscript:

MLP	Multilayer perceptron
IMU	Inertial measurement unit
EMG	Electromyograph
W	Walking
RA	Ramp ascent
RD	Ramp descent
SA	Stair ascent
SD	Stair descent
O	Obstacle
FC	Foot contact
TO	Toe off
